# Abortion-related complications in Brazil: results from the World Health Organization *Multi-country Survey on Abortion* (MCS-A)

**DOI:** 10.1590/0102-311XEN010624

**Published:** 2025-01-13

**Authors:** Nelio Neves Veiga, Luiz Francisco Cintra Baccaro, MCS-A Brazil Collaboration Group 2, Adriana Mendonça da Silva Alexandrino, Alexandre Volta Andrade do Nascimento, Camila Tereza Camilo Clerot, Cintya Andreia do Nascimento Santos, Claudia Lucrécia de Matos Silva, Claudio Lucio de Medeiros Albuquerque, Débora Paulo Santos, Demétrio Antonio Gonçalves da S. Gomes, Édson Cunha de Araújo, Elizabete das Chagas Vilanova, Georgina Costa Teixeira, Graciete Helena Nascimento dos Santos, Guilherme Augusto Guerra Avelar, Iara Elce Lopes Barros, Jaqueline Polon Abboud, Jeane Cristina Antas Lins, Joanne Thalita Pereira Silva, Kelma H. Aguiar de Lucena, Lara Wanderley Paes Barbosa, Lia Ferreira Caixeta Barreto de Siqueira, Luciana de Melo Freitas Carneiro, Lucila Nagata, Márcia de Oliveira Pereira Lúcio, Maria Cleane Rodrigues da Silva, Maria da Conceição Ribeiro Simões, Maria Fernanda da Mota Magalhães, Mariana Viana Almeida, Michelle Regina Faria Lira, Nádia Martins de Paula Souza, Rafael Martins Papa, Rafael Ribeiro Matos, Renata Porto Pinheiro, Roberta Souza dos Anjos, Suelen Miranda de Jesus, Tedy Roger Flores Ynturias, Valéria Cristina Gonçalves, Viviane Resende de Abreu Caetano

**Affiliations:** 1 Universidade Estadual de Campinas, Campinas, Brasil.; 2 Escola de Ciências da Saúde, Sobradinho, Brasil; 3 Hospital Regional de Barra do Corda, Barra do Corda, Brasil; 4 Hospital Regional de Taguatinga, Taguatinga, Brasil; 5 Hospital Universitário, Universidade Federal do Maranhão, São Luís, Brasil; 6 Secretaria Municipal da Saúde de Vilhena, Vilhena, Brasil; 7 Secretaria de Saúde do Distrito Federal, Brasília, Brasil; 8 Universidade Católica de Brasília, Brasília, Brasil; 9 Maternidade de Alta Complexidade do Maranhão, São Luís, Brasil; 10 Hospital Regional de Samambaia, Samambaia, Brasil; 11 Maternidade Paço do Lumiar, Paço do Lumiar, Brasil; 12 Maternidade Mãe Esperança, Porto Velho, Brasil; 13 Hospital Regional da Ceilândia, Ceilândia, Brasil; 14 Hospital Universitário Ana Bezerra, Universidade Federal do Rio Grande do Norte, Natal, Brasil; 15 Maternidade Benedito Leite, São Luís, Brasil; 16 Hospital Materno Infantil de Brasília, Brasília, Brasil; 17 Municipal de Saúde de Ji-Paraná, Ji-Paraná, Brasil; 18 Hospital Santa Casa de Misericórdia do Maranhão, São Luís, Brasil; 19 Centro Universitário São Lucas, Porto Velho, Brasil; 20 Hospital Regional de Sobradinho, Sobradinho, Brasil; 21 Hospital Regional Adamastor Teixeira de Oliveira, Vilhena, Brasil; 22 Hospital Regional Materno Infantil de Imperatriz, Imperatriz, Brasil

**Keywords:** Abortion, Induced Abortion, Incomplete Abortion, Missed Abortion, Septic Abortion, Aborto, Aborto Induzido, Aborto Incompleto, Aborto Retido, Aborto Séptico, VIH, Profilaxia Pre-Exposición, Adolescent, Tecnología para la Salud

## Abstract

This study aimed to describe the severity of abortion-related complications, factors associated with complications, the types of management and the experience of care in Brazil. A cross-sectional study in twenty hospitals (10 in Federal District, 3 in Rondônia and 7 in Maranhão). For 3 months, all women treated for abortion/miscarriage had their data collected. The severity of complications was defined according to World Health Organization criteria. Women with hemorrhage, infection or organs injury were invited to answer an interview about experience of care. Statistical analysis was performed using chi-square test and Poisson regression models. Among 1,683 women included, 82.5% had mild complications, 13.6% had moderate complications, 3.2% had potentially life-threatening conditions (PLTC) and 0.7% had severe maternal outcomes (SMO). Most women (94.2%) required uterine evacuation. Among these, 91.5% required surgical evacuation (with or without the use of uterotonics) and 8.5% used only uterotonics. The most frequent surgical evacuation method was curettage (66.9%), followed by manual vacuum aspiration (MVA) (32.3%). Factors associated with PLTC/SMO vs mild complications were having a gestational age ≥ 13 weeks (pravlence ratio - PR = 3.09; 95% confidence interval - 95%CI: 1.42-6.72), having been treated in Maranhão (PR = 0.27; 95%CI: 0.12-0.63) and in Rondônia (PR = 0.64; 95%CI: 0.20-0.99). Factors associated with moderate vs. mild complications were expulsion of products of conception before arrival to health facility (PR = 2.55; 95%CI: 1.64-3.96) and having been treated in Maranhão (PR = 0.58; 95%CI: 0.38-0.87). Most women who responded to the interview were treated kindly (95.6%), however, 66.7% felt stressed and 10.1% reported that their preferences were not respected during hospitalization. Nine out of ten women treated in Brazilian public hospitals due to abortion-related complications undergo some surgical procedure, the most common of which is uterine curettage. Approximately four in every hundred women experience severe complications. It is essential to ensure the supply of equipment for MVA and to encourage continuing medical education programs to increase the awareness of healthcare professionals about safer treatments for uterine evacuation.

## Resumo

O objetivo foi descrever a gravidade das complicações relacionadas ao aborto, os fatores relacionados às complicações, os tipos de tratamento e a experiência de atendimento no Brasil. Foi realizado um estudo transversal em vinte hospitais (dez no Distrito Federal, três em Rondônia e sete no Maranhão). Durante três meses, todos os dados de todas as mulheres tratadas por aborto/aborto espontâneo foram coletados. A gravidade das complicações foi definida de acordo com os critérios da Organização Mundial da Saúde. As mulheres com hemorragia, infecção ou lesão de órgãos foram convidadas a responder a uma entrevista sobre a experiência do atendimento. A análise estatística foi realizada usando o teste de qui-quadrado e modelos de regressão de Poisson. Entre as 1.683 mulheres incluídas, 82,5% tiveram complicações leves, 13,6% tiveram complicações moderadas, 3,2% tiveram condições potencialmente ameaçadoras à vida (PLTC, acrônimo em inglês) e 0,7% tiveram resultados maternos graves (SMO, acrônimo em inglês). A maioria das mulheres (94,2%) precisou de esvaziamento uterino. Entre elas, 91,5% precisaram de esvaziamento cirúrgico (com ou sem uso de uterotônicos) e 8,5% usaram apenas uterotônicos. O método de esvaziamento cirúrgico mais frequente foi a curetagem (66,9%), seguido pela aspiração manual a vácuo (32,3%). Os fatores associados à PLTC/SMO *vs*. complicações leves foram ter idade gestacional ≥ 13 semanas (razão de prevalência - RP = 3,09; intevalo de 95% de confiança - IC95%: 1,42-6,72), ter sido tratado no Maranhão (RP = 0,27; IC95%: 0,12-0,63) e em Rondônia (RP = 0,64; IC95%: 0,20-0,99). Os fatores associados às complicações moderadas *vs*. leves foram expulsão dos produtos da concepção antes da chegada ao serviço de saúde (RP = 2,55; IC95%: 1,64-3,96) e ter sido tratado no Maranhão (RP = 0,58; IC95%: 0,38-0,87). A maioria das mulheres que responderam à entrevista foi tratada com gentileza (95,6%), no entanto, 66,7% se sentiram estressadas e 10,1% relataram que suas preferências não foram respeitadas durante a internação. Nove em cada dez mulheres atendidas em hospitais públicos brasileiros devido a complicações relacionadas ao aborto são submetidas a algum procedimento cirúrgico, sendo o mais comum a curetagem uterina. Aproximadamente quatro em cada 100 mulheres apresentam complicações graves. É fundamental garantir o fornecimento de equipamentos para aspiração manual a vácuo e incentivar programas de educação médica continuada para aumentar a conscientização dos profissionais de saúde sobre tratamentos mais seguros para evacuação uterina.

## Resumen

El objetivo de este estudio fue describir la gravedad de las complicaciones relacionadas con el aborto, los factores asociados a las complicaciones, los tipos de tratamiento y la experiencia de la atención en Brasil. Se realizó un estudio transversal en veinte hospitales (diez en el Distrito Federal, tres en Rondônia y siete en Maranhão). Durante tres meses, se recopilaron los datos de las mujeres tratadas por aborto espontáneo/aborto espontáneo. La gravedad de las complicaciones se basó en los criterios de la Organización Mundial de la Salud. Las mujeres con hemorragia, infección o daño en órganos recibieron una invitación para participar en una entrevista sobre la experiencia de atención. El análisis estadístico se realizó con la prueba de chi-cuadrado y modelos de regresión de Poisson. Entre las 1.683 mujeres participantes, el 82,5% de ellas tuvo complicaciones leves, el 13,6% complicaciones moderadas, el 3,2% afecciones potencialmente mortales (PLTC, por sus siglas en inglés) y el 0,7% tuvo resultados maternos graves (SMO, por sus siglas en inglés). La mayoría de las mujeres (94,2%) necesitaron vaciamiento uterino. Entre estas, el 91,5% requirió vaciado quirúrgico (con o sin uso de uterotónicos) y el 8,5% utilizó solo uterotónicos. La técnica de vaciamiento quirúrgico más frecuente fue el legrado (66,9%), seguido de la aspiración manual por vacío (32,3%). Los factores asociados con PLTC/SMO *vs*. complicaciones leves fueron tener edad gestacional ≥ 13 semanas (razón de prevalencia - RP = 3,09; intevalo de 95% de confianza - IC95%: 1,42-6,72), haber sido tratado en Maranhão (RP = 0,27; IC95%: 0,12-0,63) y en Rondônia (RP = 0,64; IC95%: 0,20-0,99). Los factores asociados con complicaciones moderadas *vs.* leves fueron la expulsión de productos de la concepción antes de llegar al establecimiento de salud (RP = 2,55; IC95%: 1,64-3,96) y haber sido tratado en Maranhão (RP = 0,58; IC95%: 0,38-0,87). Aunque la mayoría de las mujeres entrevistadas reportaron haber sido tratadas con amabilidad (95,6%), el 66,7% de ellas se sintieron estresadas y el 10,1% informó que sus preferencias no fueron respetadas durante la hospitalización. Nueve de cada diez mujeres atendidas en hospitales públicos brasileños por complicaciones relacionadas con el aborto se someten a algún procedimiento quirúrgico, el más común de los cuales es el legrado uterino. Aproximadamente cuatro de cada cien mujeres experimentan complicaciones graves. Es fundamental garantizar el suministro de equipos para aspiración manual por vacío y fomentar programas de educación médica continua para aumentar la conciencia de los profesionales sanitarios sobre tratamientos más seguros para la evacuación endouterina.

## Introduction

Unsafe abortion is a public health concern and one of the main causes of maternal mortality worldwide ^1^. Annually, 208 million women become pregnant around the world and almost 25% end in abortion. Moreover, almost 22 million abortions are considered unsafe, leading to 47,000 maternal deaths per year due to complications ^1^ ^,^ ^2^. Restrictive policies on access to abortion reinforce the burden of this public health issue. In Brazil, where the law ensures the right to terminate a pregnancy only in cases of sexual violence, risk of maternal death, or fetuses with anencephaly, avoidable morbidity and mortality persists ^3^ ^,^ ^4^ ^,^ ^5^. Estimates suggest that 2.4 in every 1,000 women have been hospitalized for postabortion complications and, as a consequence of unsafe abortion, 200 women die in the country every year ^4^ ^,^ ^6^. Efforts to reduce barriers and promote equity in access to safe abortion care are recommended in the World Health Organization (WHO) guidelines ^1^ ^,^ ^2^.

Brazil is the most highly populated country in Latin America and the Caribbean and shows one of the highest levels of economic and health inequalities. In the country, it is estimated that one in every seven women aged up to 40 years had at least one induced abortion, with 52% of them terminating their first pregnancy before the age of 19 ^7^. In Brazil, persistent regional disparities remain major barriers to more equitable access to health care services ^8^. This distortion is particularly acute regarding Brazil’s access to abortion and post-abortion care. The health units that perform legal abortion are located in 3.6% of municipalities and the ratio of hospitalizations from abortion varies by region. Despite the reduction of hospitalizations in the North and Northeast regions, from 2008 to 2018, their rates remained higher than in other regions of the country ^3^ ^,^ ^7^. Abortion data in legally restricted countries, such as Brazil, hinders the analysis of abortion numbers, due to underreporting and underestimation of illegal abortions. Therefore, reporting data collected in a systematic manner in this scenario is essential to assess abortion-related complications and aid in making public policy decisions ^9^ ^,^ ^10^.

The WHO *Multi-Country Survey on Abortion* (MCS-A) is a large cross-sectional study performed in 11 countries in Africa and six countries in Latin America ^11^ ^,^ ^12^ ^,^ ^13^. During three months, data on abortion-related complications were collected from women treated at health units in two states/provinces, in addition to the Federal District, in each participating country. The regional results have already been published, highlighting that the proportion of women presenting severe maternal outcomes and potentially life-threatening complications indicates that unsafe abortion continues to pose a major public health challenge. Furthermore, dilation and curettage is still widely used, despite the multiple efforts carried out by the WHO, the Pan-American Health Organization (PAHO)/Latin American Center for Perinatology (CLAP, acronym in Spanish), and the International Federation of Gynecology and Obstetrics (FIGO, acronym in French) to promote the use of safer methods of uterine evacuation, including manual vacuum aspiration (MVA) and the exclusive use of medications ^12^ ^,^ ^13^. It was also observed that a considerable portion of women do not receive explanations about the treatments carried out and that this lack of communication is even more pronounced in cases with greater morbidity ^13^ ^,^ ^14^.

Ensuring broad access to comprehensive abortion care in the health care system is crucial to meet the Sustainable Development Goals (SDGs). In 2022, Brazil showed little progress towards the 17 SDGs of the United Nations’ 2030 Agenda, with moderate improvement in gender equality and decreasing inequality ^15^. Expanding information about abortion in the country is essential, as it might support the development of targeted strategies to increase access to safe abortion and help prevent avoidable deaths. This study aimed to thoroughly describe the results of the MCS-A in Brazil, then understand the severity of abortion-related complications, factors associated with complications, types of management used, and the experience of care in 20 health units in Brazil.

## Methods

### Study design and data collection

The study protocol and specific methodological details for implementation of this large cross-sectional study are published elsewhere ^11^. Briefly, in a first stage of randomization, 11 countries in Africa and six in Latin America and the Caribbean were identified. In a second stage of randomization, two states/provinces were identified in each country, with a probability proportional to the size of the population, in addition to the Federal District. In a third stage of randomization, up to 10 health units per state were selected if they met the following inclusion criteria: > 1,000 deliveries per year, a structure with a gynecology ward, surgical capability to provide emergency obstetric care, including removal of retained products, and abortion provision and/or postabortion care. In each health facility, data were collected over a 3-month period. Trained research assistants collected data from medical records, and a representative from each health facility provided data on each hospital’s infrastructure. Data were collected from women treated for abortion-related complications (abortion/miscarriage) and who signed an informed consent form to participate. These data were related to sociodemographic information, clinical and obstetrics history, medical procedures, and health outcomes. The variables were grouped in categories. Age (≤ 19 years, 20-29 years, > 30 years), gestational age (< 13 weeks, ≥ 13 weeks), marital status (single, married/steady union, separated/divorced/widowed), educational level (no education, primary, secondary, tertiary or higher), gainful occupation (yes, no), previous births (0, ≥ 1), expulsion of products of conception (POC) before arrival to health facility (yes, no), state (Rondônia, Maranhão, Federal District), surgical uterine evacuation (yes, no), type of surgical evacuation (MVA, curettage), use of uterotonics (yes, no), use of misoprostol (yes, no), use of oxytocin (yes, no), hysterectomy (yes, no), use of intravenous fluids (yes, no), use of vasopressors (yes, no), use of procoagulant agents (yes, no), blood transfusion (yes, no), and intensive care unit admission (ICU) (yes, no).

The women who had a hospital stay for at least 24 hours and had one or more complications (infection, severe hemorrhage/anemia, injury to reproductive organs, hysterectomy/laparotomy) were eligible and invited to participate in the exit survey using audio computer-assisted self-interviews (ACASI). For the present analysis, ACASI interview questions related to experience of care were employed. All ACASI interview questions included in this analysis had a yes or no response option. The complete list of questions contained in the ACASI interview can be found as Supplementary Material (Box S1; https://cadernos.ensp.fiocruz.br/static//arquivo/supl-e00010624_5299.pdf).

In this study, data specifically collected in Brazil were analyzed, which was one of the countries selected to participate in the MCS-A in Latin America. The states selected to participate were Maranhão and Rondônia, as well as the Federal District. After randomization, the health units that met the inclusion criteria (seven hospitals in Maranhão, three hospitals in Rondônia, and 10 hospitals in the Federal District) were identified. The full list of participating institutions can be found in the Supplementary Material (Box S2; https://cadernos.ensp.fiocruz.br/static//arquivo/supl-e00010624_5299.pdf). Data were collected from September 2018 to February 2019. All women signed an informed consent form before their data were included, which was also obtained for the ACASI exit survey. The WHO’s Research Ethical Review Committee (protocol: 0002699), Research Ethical Review Committee University of Campinas (UNICAMP), the Brazilian Ethics Research Committee (CONEP), and each local Research Ethics Committee provided ethical approval of the study protocol (CAAE: 78745317.1.0000.5404).

### Statistical analysis

First, descriptive analyses of the data were generated. Continuous variables were assessed as mean ± standard deviation (SD), median (range), and quartiles. Categorical variables were assessed as relative frequencies. The classification of abortion-related complications was carried out based on clinical signs, symptoms, and laboratory tests, following the WHO criteria for near miss ^16^. Women were divided into mild complications, based on abnormal physical examination findings on initial assessment; moderate complications, including heavy bleeding, suspected intra-abdominal injury, or infection; potentially life-threatening complications (PLTC), including severe hemorrhage, severe systemic infection or suspected uterine perforation); and severe maternal outcomes (SMO), based on WHO maternal near miss criteria, including organ dysfunction of either one or more of the following: cardiovascular, respiratory, renal, coagulation, hepatic, neurologic, or uterine dysfunction, as well as maternal deaths.

All variables were analyzed according to the severity of the complications using the chi-square test or Fisher’s exact test. To evaluate factors associated with abortion-related complications, two Poisson regression models were constructed, one comparing SMO+PLTC versus mild complications and another model comparing mild complications versus moderate complications. The models were adjusted for age, marital status, number of previous births, gestational age, expulsion of ovular remains before arrival at the hospital, and federative unit of Brazil. Statistical analysis was performed using the SAS program (https://www.sas.com/), version 9.2.

## Results

We analyzed data from 1,683 women with abortion-related complications, 789 in Maranhão (46.9%), 728 (43.3%) in the Federal District, and 166 (9.8%) in Rondônia. From all women who had abortion-related complications, 1,389 (82.5%) had mild complications, 228 (13.6%) had moderate complications, 54 (3.2%) had PLTC, and 12 (0.7%) had SMO, being 12 cases of near miss and no deaths. Table 1 shows the criteria for hierarchal classification of abortion-related complications. Among these women, 1,487 (86.7%) were over 20 years old, 705 (49%) were single, and 1,052 (83%) had completed secondary or tertiary education. Most women (91.6%) had previous births, gestational age less than 13 weeks (83.2%) and did not expel POC before arrival at the health unit (83.2%). We highlight that patients with gestational age ≥ 13 weeks had a higher prevalence of PLTC/SMO (p < 0.01), those who expelled POC before arrival at the health unit had a higher prevalence of moderate complications and SMO (p < 0.01), and those treated in Maranhão and in Rondônia had a higher prevalence of mild complications (p < 0.01). Table 2 describes sociodemographic and obstetrics characteristics by severity of abortion-related complications. The factors independently associated with the occurrence of PLTC/SMO were having a gestational age ≥ 13 weeks (prevalence ratio - PR = 3.09; 95% confidence interval - 95%CI: 1.42-6.72), having been treated in Maranhão (PR = 0.27; 95%CI: 0.12-0.63) and in Rondônia (PR = 0.64; 95%CI: 0.20-0.99). Expulsion of POC before arrival to the health facility (PR = 2.55; 95%CI: 1.64-3.96) and being treated in Maranhão (PR = 0.58; 95%CI: 0.38-0.87) were associated with moderate complications in comparison with mild complications (Table 3).

**Table 1:** Criteria for hierarchal classification of abortion-related complications * - World Health Organization (WHO) *Multi-Country Survey on Abortion* (MCS-A).

Variables	n	%
Mild **	1,389	82.53
Vaginal bleeding	1,345	79.90
Cervix open	648	41.80
Uterine tenderness	143	8.50
Cervical motion tenderness	114	6.80
Foul smelling vaginal discharge	76	4.50
Moderate ***	228	13.55
Heavy bleeding	225	13.40
Suspected intra-abdominal injury	47	2.80
Infection	63	3.70
PTLC ^#^	54	3.21
Severe hemorrhage	33	2.00
Severe systemic infection	32	1.90
Uterine perforation	0	0.00
SMO ^##^	12	0.71
Cardiovascular	4	0.20
Coagulation	4	0.20
Neurologic	1	0.10
Hepatic	1	0.10
Uterine	4	0.20
Renal	3	0.18
Respiratory	2	0.12

PLTC: potentially life threatening complications; SMO: severe maternal outcomes.

* Women may have had more than one complication;

** Mild complications based on abnormal physical examination findings on initial assessment;

*** Moderate complications (heavy bleeding, suspected intra-abdominal injury, or infection);

^#^ WHO PLTC (severe hemorrhage, severe systemic infection, or suspected uterine perforation);

^##^ SMO according to the WHO maternal near miss criteria (organ dysfunction of either one or more of the following: cardiovascular, respiratory, renal, coagulation, hepatic, neurologic, or uterine dysfunction) and maternal deaths.

**Table 2:** Sociodemographic and obstetrical characteristics by severity of abortion-related complications (N =1,683).

Characteristics	Total	Mild *	Moderate **	PLTC ***	SMO ^#^
	n (%)	n (%)	n (%)	n (%)	n (%)
Age (years) ^a^					
≤ 19	224 (13.3)	182 (13.1)	33 (14.5)	7 (13.0)	2 (16.7)
20-29	770 (45.8)	625 (45.0)	117 (51.5)	21 (38.9)	7 (58.3)
≥ 30	687 (40.9)	581 (41.9)	77 (33.9)	26 (48.1)	3 (25.0)
Marital status ^b^					
With partner	697 (48.5)	581 (48.2)	89 (50.3)	22 (50.0)	5 (50.0)
Without partner	740 (51.5)	625 (51.8)	88 (49.7)	22 (50.0)	5 (50.0)
Educational level ^c^					
No education	6 (0.5)	3 (0.3)	3 (2.1)	0 (0.0)	0 (0.0)
Primary	209 (16.5)	179 (16.5)	22 (15.6)	5 (14.7)	3 (33.3)
Secondary	878 (69.3)	748 (69.1)	103 (73.1)	21 (61.8)	6 (66.7)
Tertiary or higher	174 (13.7)	153 (14.1)	13 (9.2)	8 (23.5)	0 (0.0)
Gainful occupation ^d^					
Yes	514 (38.8)	426 (37.8)	61 (41.8)	23 (57.5)	4 (36.4)
No	809 (61.2)	700 (62.2)	85 (58.2)	17 (42.5)	7 (63.6)
Previous births ^e^					
≥ 1	1,078 (91.7)	892 (91.3)	141 (92.8)	36 (94.7)	9 (100.0)
0	98 (8.3)	85 (8.7)	11 (7.2)	2 (5.3)	0 (0.0)
Previous abortions ^f^					
≥ 1	417 (35.6)	344 (35.3)	49 (32.2)	21 (55.3)	3 (33.3)
0	756 (64.4)	630 (64.7)	103 (67.8)	17 (44.7)	6 (66.7)
Gestational age (weeks) ^g^					
< 13	1,266 (83.2)	1,089 (84.9) ^##^	149 (80.1) ^##^	22 (52.4)	6 (60.0)
≥ 13	255 (16.8)	194 (15.1)	37 (19.9)	20 (47.6) ^##^	4 (40.0) ^##^
Expulsion of POC before arrival at the facility ^h^					
No	1,374 (83.2)	1,166 (85.4) ^##^	158 (71.2)	41 (78.9) ^##^	9 (75.0)
Yes	277 (16.8)	199 (14.6)	64 (28.8) ^##^	11 (21.1)	3 (25.0) ^##^
Federative Unit					
Federal District	728 (43.3)	537 (38.7)	142 (62.3) ^##^	41 (76.0) ^##^	8 (66.7) ^##^
Maranhão	789 (46.9)	703 (50.6) ^##^	70 (30.7)	12 (22.2)	4 (33.3)
Rondônia	166 (9.8)	149 (10.7) ^##^	16 (7.0)	1 (1.8)	0 (0.0)

PLTC: potentially life threatening complications; POC: products of conception; SMO: severe maternal outcomes.

Note: missing cases: a = 2, b = 246, c = 416, d = 360, e = 507, f = 510, g = 162, h = 32.

* Mild complications based on abnormal physical examination findings on initial assessment;

** Moderate complications (heavy bleeding, suspected intra-abdominal injury, or infection);

*** World Health Organization (WHO) PLTC (severe hemorrhage, severe systemic infection, or suspected uterine perforation);

^#^ SMO according to the WHO maternal near miss criteria (organ dysfunction of either one or more of the following: cardiovascular, respiratory, renal, coagulation, hepatic, neurologic, or uterine dysfunction) and maternal deaths;

^##^ p-value < 0.001, chi-square test.

**Table 3:** Factors associated with abortion-related complications - Poisson regression model.

Factor/Categories	PLTC/SMO vs. mild *			Moderate vs. mild **		
	p-value	PR	95%CI	p-value	PR	95%CI
Age (years)						
≤ 19 (Reference)	-	1.00	-	-	1.00	-
20-29	0.86	1.20	0.15-9.34	0.10	0.55	0.27-1.13
≥ 30	0.78	1.32	0.17-10.08	0.07	0.52	0.25-1.07
Marital status						
With partner (Reference)	-	1.00	-	-	1.00	-
Without partner	0.16	0.55	0.24-1.28	0.71	0.93	0.62-1.40
Number of births						
0 (Reference)	-	1.00	-	-	1.00	-
≥ 1	0.54	1.57	0.37-6.67	0.20	1.73	0.75-3.98
Gestational age (weeks)						
< 13 (Reference)	-	1.00	-	-	1.00	-
≥ 13	< 0.01	3.09	1.42-6.72	0.25	1.34	0.81-2.22
Expulsion of POC before arrival						
No (Reference)	-	1.00	-	-	1.00	-
Yes	0.99	1.01	0.30-3.35	< 0.01	2.55	1.64-3.96
Federative Unit						
Federal District (Reference)	-	1.00	-	-	1.00	-
Maranhão	< 0.01	0.27	0.12-0.63	0.01	0.58	0.38-0.87
Rondônia	0.04	0.64	0.20-0.99	0.09	0.53	0.25-1.11

95%CI: 95% confidence interval; PLTC: potentially life threatening complications; POC: products of conception; PR: prevalence ratio; SMO: severe maternal outcomes.

* Poisson regression model: n = 788 mild, and n = 27 PLTC/SMO;

** Poisson regression model: n = 788 mild, and n = 99 moderate.

Among all participants, 1,586 (94.2%) required some type of intervention for uterine evacuation. Among these, 1,451 (91.5%) required surgical uterine evacuation and 135 (8.5%) used only uterotonic medications. Among women who underwent surgical uterine evacuation, 1,021 (70.4%) also received uterotonic medications. Of the women who underwent surgical evacuation and received uterotonics, 296 received single dose misoprostol (29%). The most frequently used surgical evacuation method was uterine curettage (66.9%), followed by manual intrauterine aspiration (32.3%). In total, 506 women (43.8%) received misoprostol alone, 156 (13.5%) received oxytocin alone, 154 (13.3%) received combined misoprostol/oxytocin, and 174 (15%) received ergometrine alone. Among the other supportive treatment modalities used, 1,239 (73.6%) received intravenous fluids, 5 (0.3%) received vasopressor agents, 401 (23.8%) received antibiotics, 9 (0.5%) received procoagulant agents, 22 (1.3%) received a blood transfusion, 3 (0.2%) underwent exploratory laparotomy, 1 (0.01%) underwent hysterectomy, and 4 (0.2%) were treated in an ICU. The use of misoprostol was higher among women with mild complications (73.7%), and the use of oxytocin (alone or combined with misoprostol) was more frequent in those with severe outcomes (75%). Women with severe complications received more intravenous fluids (91.7%), vasopressors (16.7%), antibiotics (50%), procoagulants (8.3%), blood transfusions (33.3%), and were admitted to the ICU more often (25%). Table 4 details the different types of treatment used for women according to the severity of complications.

**Table 4:** Types of management according to severity of complications.

Type of management	Mild	Moderate	PLTC	SMO	p-value *
	n (%)	n (%)	n (%)	n (%)	
Surgical uterine evacuation					0.72
Yes	1,199 (86.3)	197 (86.4)	46 (85.2)	9 (75.0)	
No	190 (13.7)	31 (13.6)	8 (14.8)	3 (25.0)	
Use of uterotonics					0.89
Yes	956 (68.8)	153 (67.1)	39 (72.2)	8 (66.7)	
No	433 (31.2)	75 (32.9)	15 (27.8)	4 (33.3)	
Misoprostol					< 0.01
Yes	705 (73.7)	55 (36.0)	16 (41.0)	4 (50.0)	
No	251 (26.3)	98 (64.0)	23 (59.0)	4 (50.0)	
Oxytocin					< 0.01
Yes	262 (27.4)	84 (54.9)	21 (53.8)	6 (75.0)	
No	694 (72.6)	69 (45.1)	18 (46.2)	2 (25.0)	
Hysterectomy					< 0.01
Yes	0 (0.0)	0 (0.0)	0 (0.0)	1 (8.3)	
No	1,389 (100.0)	228 (100.0)	54 (100.0)	11 (91.7)	
Intravenous fluids					< 0.01
Yes	958 (69.0)	219 (96.0)	51 (94.4)	11 (91.7)	
No	431 (31.0)	9 (4.0)	3 (5.6)	1 (8.3)	
Vasopressors					< 0.01
Yes	1 (0.1)	0 (0.0)	2 (3.7)	2 (16.7)	
No	1,388 (99.9)	228 (100.0)	52 (96.3)	10 (83.3)	
Antibiotics					< 0.01
Yes	253 (18.2)	104 (45.6)	38 (70.4)	6 (50.0)	
No	1,136 (81.8)	124 (54.4)	16 (29.6)	6 (50.0)	
Procoagulant agents					< 0.01
Yes	2 (0.1)	3 (1.3)	3 (5.6)	1 (8.3)	
No	1,387 (99.9)	225 (98.7)	51 (94.4)	11 (91.7)	
Blood transfusion					< 0.01
Yes	0 (0.0)	0 (0.0)	18 (33.3)	4 (33.3)	
No	1,389 (100.0)	228 (100.0)	36 (66.7)	8 (66.7)	
ICU admission					< 0.01
Yes	0 (0.0)	0 (0.0)	1 (1.8)	3 (25.0)	
No	1,389 (100.0)	228 (100.0)	53 (98.2)	9 (75.0)	

ICU: intensive care unit admission; PLTC: potentially life threatening complications; SMO: severe maternal outcomes.

* Chi-square test.

In total, 70 women participated in the ACASI interview; 66 (95.6%) reported having received explanations about the treatment they received, 66 (95.6%) reported having been treated kindly and 65 (94.2%) were able to ask questions during the examination/treatment. However, 9 (13%) women reported that the professional did not explain everything they needed to know about the treatment and 7 (10.1%) women reported that their preferences about the treatment were not respected. Anxiety/stress during hospitalization was reported by 46 (66.7%) women. Among them, 12 (26.1%) were unable to communicate this feeling to the health professional. Among the 34 women who spoke about anxiety/stress with the health professional, 13 (38.2%) reported that the professional did not offer any help to alleviate the symptom. Table 5 details the responses to the ACASI questionnaire according to the severity of the complications. We highlight that women with mild complications who requested additional help to relieve anxiety/stress did not receive it (p < 0.05).

**Table 5:** Women’s experience of care: audio computer-assisted self-interviews (ACASI) answers by severity (N = 70).

Questions	Total n (%)	Severity of abortion-related complications			
		Mild	Moderate	PLTC	SMO
		n (%)	n (%)	n (%)	n (%)
During your stay at this hospital, were you given explanations regarding your care and treatment? *					
Yes	66 (95.6)	11 (100.0)	30 (93.8)	21 (95.4)	4 (100.0)
No	3 (4.4)	0 (0.0)	2 (6.2)	1 (4.6)	0 (0.0)
Were you able to ask questions during the examination and treatment? *					
Yes	65 (94.2)	11 (100.0)	30 (93.8)	20 (90.9)	4 (100.0)
No	4 (5.8)	0 (0.0)	2 (6.2)	2 (9.1)	0 (0.0)
Do you feel your health care provider told you everything you need to know about decisions taken for your care? *					
Yes	60 (86.9)	9 (81.8)	30 (93.8)	18 (81.8)	3 (75)
No	9 (13.1)	2 (18.2)	2 (6.2)	4 (18.2)	1 (25)
Did you encounter any anxiety or stress during your hospital stay? *					
Yes	46 (66.6)	8 (72.7)	21 (65.6)	14 (63.6)	3 (75)
No	23 (33.4)	3 (27.3)	11 (34.4)	8 (36.4)	1 (25)
When you told the physician or nurse about feeling the anxiety or stress, did he or she offer additional support to help ease your anxiety or stress? **^,^***					
Yes	21 (61.8)	0 (0.0)	11 (73.3)	8 (66.7)	2 (100.0)
No	13 (38.2)	5 (100.0)	4 (26.7)	4 (33.3)	0 (0.0)
Were you spoken to nicely?					
Yes	66 (95.6)	11 (100.0)	32 (100.0)	19 (86.4)	4 (100.0)
No	3 (4.4)	0 (0.0)	0 (0.0)	3 (13.6)	0 (0.0)

PLTC: potentially life threatening complications; SMO: severe maternal outcomes.

* Missing: 1;

** Missing: 12;

*** Fisher’s exact test, p < 0.05.

## Discussion

This study described, using standardized methodology, the severity of abortion-related complications, factors associated with complications, type of management, and experience of care of women across 20 hospitals located in three Brazilian’s Federative Units. Expanding knowledge on the topic in a country with diverse socioeconomic contexts is essential for the development of public health policies aimed at reducing morbidity and mortality related to abortion. The results showed that most women presented mild complications. The factor associated with PLTC/SMO versus mild complications was having a gestational age ≥ 13 weeks. The factor associated with moderate versus mild complications was expulsion of products of conception before arrival to health facility. Women treated in Maranhão and Rondônia had a lower prevalence of serious complications when compared to women in the Federal District. The use of non-recommended uterine evacuation methods, including curettage and double method, remains prevalent. When evaluating the experience of care, despite women reporting kindness during treatment, anxiety and stress are still present.

Since 2012, the use of a standardized classification for complications associated with pregnancy has allowed the comparison of different contexts in different regions ^17^. In 2013, WHO published the results of a large multi-center study using this classification to understand maternal and neonatal morbidity globally ^18^. However, in the sample of women studied, abortion-related complications were underreported, which led to the development of a new research protocol, aimed at the specific study of complications in early pregnancy ^11^. Recently, the main results of the MCS-A study in Africa and Latin America were published. An important difference was observed in the severity of abortion-related complications on the two continents. While in Africa, almost 10% of women were classified as having serious complications (2.4% of SMO and 7% of PLTC) ^12^, in Latin America less than 5% had more serious complications (1.3% of SMO and 3.1% PLTC) ^13^.

In Brazil, data from the latest research have suggested that health complications resulting from abortion are decreasing ^7^ ^,^ ^10^. In 2010, it was estimated that 55% of women who had an abortion needed to be hospitalized, and this proportion was estimated at 43% in 2021 ^17^. We observed that the prevalence of serious complications in the country was similar to other Latin American countries, as 3.2% of Brazilian women had PLTC and 0.7% had SMO. However, the number of complications classified as mild was higher in Brazil (82.5%) compared to the total of Latin American countries (46.3%). The main findings that classified the abortion complication as mild were the presence of vaginal bleeding (80%) and an open cervix (42%) on physical examination. Progressing on the severity scale, the main finding that classified the abortion complication as moderate was the presence of heavy bleeding, characterized as heavy bright red vaginal bleeding (with or without clots), blood soaked pads/towels/clothing, which occurred in 13.4% of women. PLTC cases were due to severe hemorrhage (perceived blood loss greater than 1,000mL, any bleeding with hypotension and/or requiring blood transfusion) (2%) or severe systemic infection (1.9%). We found no cases of uterine perforation. Abortion care can be classified according to its safety as safe, less-safe, or least-safe, depending on the person providing the care and the method used ^19^. Possibly, better access to health services in Brazil compared to other countries on the continent, associated with the transition from the use of rudimentary uterine evacuation methods to the use of misoprostol, even outside the formal health context, may explain these findings.

When observing factors associated with greater severity of complications, we found that having a higher gestational age and expelling POC before arrival at the health unit were associated with more severe complications. Possibly, women with a greater quantity of intrauterine material (greater gestational age) and who had a longer delay in obtaining specialized care (expulsion of POC before arriving at the hospital) presented a greater volume of blood loss, thus had more severe complications. Women treated in Maranhão and Rondônia had less severe complications compared to women treated in the Federal District. It is estimated that the demographic density of Rondônia is 6.65 inhabitants/km^2^ and the demographic density of Maranhão is 20.56 inhabitants/km^2^ ^20^. The Federal District shows an estimated demographic density of 489.06 inhabitants/km^2^ ^20^. A possible hypothesis to explain our findings is that the population density influences the occurrence of unintended pregnancies and, consequently, the number of unsafe abortions. A study carried out in the United States identified that higher demographic densities were not significantly correlated with teen pregnancy rate but were significantly correlated with the percentage of teen pregnancies that were electively terminated, possibly due to greater population access to the procedure ^21^. The authors stated that population density is likely to be a proxy for a complex set of medical, familial, religious, economic, and legal factors that influence reproductive health choices ^21^. Regional characteristics of access to health services could have also influenced our findings. According to data from the Brazilian Ministry of Health, during the data collection period of our study, the estimated population covered by the primary health care teams in the Federal District, Rondônia, and Maranhão was 61%, 73%, and 86%, respectively. The estimated population covered by the “Family Health Strategy teams” in the Federal District, Rondônia, and Maranhão was 54%, 69%, and 84%, respectively ^22^. This difference in primary health care coverage may have affected access to sexual and reproductive health in the Federal District. However, as the data collection period was relatively short (three months), it is not possible to conclude that there are substantial differences in the quality of reproductive health care provided across these Federative Units.

In recent years, international organizations have encouraged the use of less invasive methods of uterine evacuation, including manual intrauterine aspiration or uterine evacuation exclusively with medication ^1^ ^,^ ^23^ ^,^ ^24^. Curettage is a procedure associated with a greater occurrence of immediate complications, including uterine perforation ^25^, and late complications, including endometrial synechiae ^26^ ^,^ ^27^. Intrauterine manual aspiration shows a lower incidence of complications, including hemorrhage requiring blood transfusion and uterine perforations, compared to curettage ^24^ ^,^ ^28^. In this study, nine out of 10 women who sought hospital care for abortion-related complications underwent some type of medical intervention to complete uterine emptying. The data obtained confirm that uterine curettage is still widely performed in Brazil, corroborating previous data that show that this is the second most performed obstetric procedure in public hospitals in the country ^29^. Although uterine evacuation with the exclusive use of medication is considered a safe and effective procedure ^30^ ^,^ ^31^, it showed a low rate of use in this study (8%). Some factors may influence this finding. Mifepristone, a medication that is included in the uterine evacuation protocol in several countries, is not approved for use in Brazil. Therefore, medicated emptying depends on the use of misoprostol only, which can prolong the time needed to expel ovular remains. Moreover, the use of misoprostol in Brazil must be conducted exclusively in a hospital facility. A longer length of stay may justify the underuse of this method in Brazil. This hypothesis becomes clear when we analyze the double method data (medicines/surgery) obtained. We highlight that 70% of women undergoing surgical evacuation also received medication, but only 29% for cervix preparation. Possibly, women who did not use misoprostol to prepare the cervix started using misoprostol with the aim of non-surgical uterine emptying. However, with a prolonged hospitalization, surgical evacuation may have been chosen to shorten the hospital stay.

When we specifically consider the type of uterotonic, we observed that the most commonly used was misoprostol, followed by oxytocin and ergometrine. Women with milder complications received more misoprostol, and women with more severe complications received more oxytocin. As expected, the use of more complex management methods, including hysterectomy, use of vasopressors, use of procoagulants, and blood transfusion, were more frequent in women with more severe complications. However, we highlight the low number of ICU admissions. Despite the severity, only 25% of women who had SMO were admitted to intensive care beds. In 2018, according to data from the Brazilian National Registry of Health Establishments, the country presented almost 45,000 ICU beds. Less than half of these beds (49%) were available for the Brazilian Unified National Health System (SUS, acronym in Portuguese), whereas the other half were destined exclusively for private or supplementary health care, which served 23% of the population ^32^. Although the number of ICU beds has increased in recent years, the number of beds remains insufficient, especially within the SUS. According to guidelines from the WHO and the Brazilian Ministry of Health, the ideal proportion of ICU beds is one to three beds for every 10,000 inhabitants. The proportion of ICU beds available to the SUS among the federative units participating in the research was 1.01 per 10,000 in Rondônia, 0.59 per 10,000 in Maranhão, and 0.89 per 10,000 in the Federal District, underscoring a deficit in the number of ICU beds, in addition to possible issues with the management of intensive care beds ^32^.

Women-centered health care is very important in post-abortion situations. The WHO *Abortion Care Guideline* ^1^ recommends that women who arrive at health services due to abortion complications may be treated urgently and respectfully, like any other emergency patient, without punitive, judgmental, or biased attitudes. However, in African countries, 60% of women with abortion-related complications reported at least one negative experience during treatment ^14^. Treating people with kindness and ensuring that women’s preferences, needs, and values are respected can impact adverse outcomes and improve adherence to post-abortion care programs ^14^ ^,^ ^33^. In this study, most women with severe complications and eligible to respond to the hospital discharge interview (ACASI) reported having received explanations about the treatment received, were able to ask questions, and were treated with kindness. Our findings are significantly different from those found in a study conducted in Teresina (Piauí State), where one in three women reported a lack of privacy in the facility, as well as mistreatment and discrimination by providers during care for complications from an induced abortion ^34^. Possibly, the fact that our study included both spontaneous and induced abortions may explain the difference found. In our study, 1 in 10 women did not feel their choices were respected. A similar situation was described when evaluating all Latin American countries that participated in MCS-A, in which 14.1% of women did not have the opportunity to ask questions about their treatment ^13^. Anxiety and stress associated with abortion-related complications were common and can be expected in women with more severe clinical conditions. However, we highlight that approximately one in four women who felt anxious/stressed did not communicate this fact to the health team, and, in addition, among the women who commented on the subject, almost 40% did not get any type of help to improve the symptom. We highlight that no woman with mild complications who participated in the ACASI interview and who requested additional help to relieve anxiety/stress received it. Lack of emotional support can worsen the anxiety faced by women ^35^.

This study shows limitations that need to be acknowledged. Since it is a cross-sectional study, it is not possible to establish cause and effect associations. The data collection period was three continuous months in each health unit; however, the start date of collection was not the same in all hospitals. Altogether, data were collected from September 2018 to February 2019. We believe that this small difference was not capable of causing bias. Only three states in the country were included and all hospitals that met the inclusion criteria were public institutions. Consequently, the results cannot be extrapolated to the private health care system and to other states in Brazil. In the vast majority of cases, it was not possible to differentiate spontaneous abortions from induced abortions. The question in the ACASI questionnaire on anxiety/stress does not allow us to differentiate whether these emotions were caused by the hospitalization itself or whether they were already present during hospitalization, caused by the clinical condition itself. Going through the experience of abortion/miscarriage, especially when the pregnancy is intended, can bring a range of negative feelings to the woman. However, we highlight that our findings are credible due to the large number of women included, the appropriate randomization methodology of Federative Units and hospitals in the country, the detailed classification of abortion-related complications, and the rigor in data collection. Moreover, obtaining experience of care data highlighted that post-abortion care should always be patient-centered.

In total, nine out of 10 women treated in Brazilian public hospitals due to abortion complications undergo some surgical procedure, the most common of which is uterine curettage. This is possibly a reflection of the difficulty in accessing less invasive and safer techniques such as MVA or the exclusive use of misoprostol. Efforts, both by government agencies and medical societies, aimed at changing this scenario are urgent. It is essential to encourage continuing medical education programs to increase the awareness of health care professionals about safer treatments for uterine evacuation. Moreover, action towards those responsible for the administration of health facilities is essential to guarantee the acquisition of hospital supplies required for the regular implementation of MVA on a permanent basis. Approximately four in every 100 women experience severe complications, which occur more frequently in those with more advanced gestational age and who expel POC before reaching the hospital. Anxiety and stress are common in women who experience abortion-related complications. Access to post-abortion care must be broad and equitable. In a country with restrictive laws like Brazil, knowing the epidemiology of abortion-related complications is essential for formulating public policies that guarantee comprehensive care for women’s health.
