# Supplementary material for: Abortion-related complications in Brazil: results from the World Health Organization Multi-country Survey on Abortion (MCS-A)
Source: Cad Saude Publica. 2025 Jan 13;40(10):e00010624. doi: 10.1590/0102-311XEN010624 (PMC11729379; doi:10.1590/0102-311XEN010624)
Supplement: Supplementary file 1 [file 1678-4464-csp-40-10-EN010624-s.pdf]

Box S1 The complete list of questions contained in the audio computer-assisted self-interviews (ACASI).

NOME DO ARQUIVO	TEXTO A SER NARRADO
Welcome	*Bem-vinda! Por favor ajuste o volume para um nível confortável. Quando estiver pronta* por favor pressione a seta para começar.
T_01	*Obrigada pelo seu apoio neste estudo importante.*
T_02	*Antes de iniciar, vamos apresentar-lhe três perguntas de ensaio para* que você aprenda *como responder as perguntas.*
T_03	Você vai ouvir *cada uma das perguntas, e a seguir* deverá PRESSIONAR ou TOCAR em um *dos botões de resposta no meio da tela.*
T_04	*Se quiser ouvir novamente a pergunta e as respostas, PRESSIONE o botão do alto-falante que se encontra aqui.*
T_05	*Se não quiser responder a uma pergunta, PRESSIONE este botão aqui.*
T_06	*Se quiser parar com a entrevista, PRESSIONE este botão aqui.*
T_06A	*Esta barra lhe mostrará quanto da sua entrevista já está completa.*
T_07	*Depois de responder a cada pergunta, uma seta aparecerá aqui.*
T_08	*PRESSIONE esta seta para avançar para a primeira pergunta*
T_09	*Esta primeira pergunta de ensaio tem duas opções de resposta, que são: SIM e NÃO.*
T_10	Você esta pronta para começar? *Caso SIM, PRESSIONE 1. Caso NÃO, PRESSIONE 2*
T_11	*Não.*<br>*Se não estiver pronta, por favor peça ajuda.*
T_12	*Sim. Parabéns! Você respondeu a sua primeira pergunta.*<br>*Agora PRESSIONE a seta para seguir para a próxima pergunta.*
T_13	*A segunda pergunta de ensaio lhe pede para digitar um número. Por favor PRESSIONE qualquer número entre 0 e 9.*
T_14	*Ótimo! Agora PRESSIONE a seta para avançar para a próxima pergunta.*
T_15	*A terceira pergunta do ensaio pergunta-lhe sobre o seu nível de satisfação. Esta pergunta tem 5 opções de resposta, que são: Muito satisfeita ... Satisfeita… Nem satisfeita, nem insatisfeita… Insatisfeita… Muito insatisfeita*
T_16	*Qual é o seu nível de satisfação com o seu sono ontem à noite?*<br>*Para muito satisfeita, PRESSIONE 1.*<br>*Para satisfeita, PRESSIONE 2*<br>*Para nem satisfeita, nem insatisfeita, PRESSIONE 3*<br>*Para insatisfeita, PRESSIONE 4*<br>*Para muito Insatisfeita, PRESSIONE 5*
T_17_01_VS	*Muito satisfeita.*<br>*Muito bem! Você respondeu as 3 perguntas do ensaio.*<br>*Agora PRESSIONE a seta para iniciar.*
T_17_02_S	*Satisfeita.*<br>*Muito bem! Você respondeu as 3 perguntas do ensaio.*<br>*Agora PRESSIONE a seta para iniciar.*
T_17_03_N	*Nem satisfeita, nem insatisfeita.*<br>*Muito bem! Você respondeu as 3 perguntas do ensaio.*<br>*Agora PRESSIONE a seta para iniciar.*
T_17_04_D	*Insatisfeita.*<br>*Muito bem! Você respondeu as 3 perguntas do ensaio.*<br>*Agora PRESSIONE a seta para iniciar.*
T_17_05_VD	*Muito insatisfeita.*<br>*Muito bem! Você respondeu as 3 perguntas do ensaio.*<br>*Agora PRESSIONE a seta para iniciar.*
PRIVACY	Agora iniciaremos as perguntas da entrevista. *Agradecemos as suas respostas e queremos relembrar que todas as perguntas são confidenciais.*<br>*O sistema usado para esta entrevista protege a sua privacidade.*
INTRO_101	*As próximas perguntas são sobre o seu padrão de vida.*<br>*Por favor escute cada pergunta e responda SIM ou NÃO.*
Q_101	*Você tem água encanada na sua casa?*
Q_102	*Durante o último mês, o seu rendimento familiar foi suficiente para cobrir todas a suas necessidades pessoais, tais como comida e saúde?*
Q_103	*Depois de pagar as despesas mensais da casa, algum membro da sua família conseguiu poupar algum dinheiro durante o mês passado?*
Q_104	*Durante o último mês, alguma vez tiveram que passar um dia inteiro sem nenhuma refeição devido à falta de comida ou de dinheiro para comprar comida?*
Q_105	*Durante o último mês, você ganhou o seu próprio dinheiro?*
Q_106	*Durante o último mês, você estudou em tempo integral?*
INTRO_107	*As próximas perguntas são sobre as suas gravidezes.*
Q_107	Você já teve algum parto *antes desta gravidez?*
Q_108	Você já teve alguma interrupção da gravidez, também chamada de aborto?

Q_109	*Quantos abortos já teve?*
Q_110	Você estava de quantos meses quando perdeu a atual gravidez?
Q_111	Quando você engravidou, você queria ficar grávida?
Q_112	Você usou algum método anticoncepcional para prevenir a gravidez?
INTRO_113	*Existem muitas razões que fazem com que uma mulher perca uma gravidez.*<br>*Por exemplo, uma mulher pode perder uma gravidez depois de uma queda, depois de tomar algum medicamento, ou depois de alguém lhe bater.* Ou, simplesmente, pode-se não saber a razão.<br>Por favor, nos diga qual você acha que foi a principal razão para que você perdesse a gravidez.<br>*Lembre-se que as suas respostas são todas confidenciais e anônimas.*
Q_113	*Você caiu de barriga ou teve uma queda grave de lado ou de costas?*
Q_114	*Você tomou algum medicamento?*
Q_115	Você tomou, ou inseriu na vagina, alguma preparação a base de ervas?
Q_116	*O seu marido ou parceiro ou outra pessoa lhe bateu?*
Q_117	*A perda da sua gravidez aconteceu de repente sem aviso?*
Q_118	*Aconteceu alguma outra coisa que não foi mencionada acima?*
INTRO_119	*Estamos interessados em saber a principal razão que lhe levou a vir ao hospital.* Pode ser que você tenha vindo porque estava com febre, dor, corrimento ou sangramento vaginal. Ou, pode ser que você tenha vindo para realizar um procedimento para limpeza do útero, ou para confirmar que o aborto foi completo. *Gostaríamos de saber o principal motivo que lhe levou a vir ao hospital.*
Q_119	*A razão principal de vir ao hospital foi porque estava com* febre, dor, corrimento ou sangramento vaginal?
Q_120	*A razão principal de vir ao hospital foi* para realizar o procedimento de limpeza do útero?
Q_121	*A razão principal de vir ao hospital foi* para confirmar que o aborto foi completo?
INTRO_122	*As seguintes perguntas são para saber mais sobre a perda da sua gravidez. Lembre-se que todas as suas respostas são confidenciais. É comum uma mulher engravidar sem querer. É também comum uma mulher decidir interromper a gravidez.*<br>*Existem várias formas de interromper uma gravidez, seja com medicamentos, ervas, inserção de algo na vagina ou procedimento cirúrgico.*
Q_122	*Você usou algum método para interromper a sua gravidez?*
INTRO_123	Gostaríamos de saber qual método ou métodos você usou para interromper a sua gravidez. *As perguntas seguintes são sobre maneiras que você pode ter usado para interromper a sua gravidez.*
Q_123	Você usou o misoprostol, também chamado de citotec, que é um comprimido para provocar o aborto, que pode ser tomado ou colocado na vagina?
Q_124	Você usou dois tipos diferentes de comprimidos?
Q_125	Você utilizou alguma outra medicação, tanto tomando pela boca, ou introduzindo na vagina?
Q_126	Você tomou pela boca ervas, alguma substância, ou uma combinação de substâncias? Por exemplo, medicações contra malária como cloroquina ou quinina, alvejante, gasolina, ou detergente?
Q_127	Você inseriu alguma coisa na vagina?
Q_128	Você realizou algum procedimento para limpar o conteúdo do seu útero?
Q_129	Você utilizou alguma massagem abdominal tradicional?
Q_130	Você usou alguma outra coisa para interromper a gravidez?
INTRO_131	*As vezes as pessoas obtêm informações sobre como interromper uma gravidez com outras pessoas, como membros da família, amigos ou farmacêuticos, ou*<br>*por meio da rádio, televisão, internet ou outros locais.*<br>*Gostaríamos de saber como você obteve informação sobre como interromper esta gravidez.*
Q_131	*Você obteve informação sobre que método usar para interromper a sua gravidez de alguém?*
Q_132	*Você obteve informação sobre o método para interromper sua gravidez do seu* ***marido, namorado ou companheiro?***
Q_133	*Você obteve a informação sobre o método para interromper sua gravidez através de* ***outras pessoas da sua família?***
Q_134	*Você obteve a informação sobre o método para interromper sua gravidez através de* ***um amigo ou uma amiga?***
Q_135	*Você obteve a informação sobre o método para interromper sua gravidez pela* ***internet ou redes sociais?***
Q_136	*Você obteve a informação sobre o método para interromper sua gravidez pela* ***rádio ou televisão?***
Q_137	*Você obteve a informação sobre o método para interromper sua gravidez através de um* ***médico?***
Q_138	*Você obteve a informação sobre o método para interromper sua gravidez através de uma* ***enfermeira ou parteira?***
Q_139	*Você obteve a informação sobre o método para interromper sua gravidez através de* ***um farmacêutico?***
Q_140	*Você obteve a informação sobre o método para interromper sua gravidez através de* ***um comerciante?***
Q_141	*Você obteve a informação sobre o método para interromper sua gravidez através de uma* ***parteira tradicional?***
Q_142	*Você obteve a informação sobre o método para interromper sua gravidez através de um* ***trabalhador de saúde comunitário?***
Q_143	*Você obteve a informação sobre o método para interromper sua gravidez através de* ***uma outra pessoa?***
INTRO_144	*Às vezes as mulheres têm alguém que lhes ajuda a interromper uma gravidez, como um médico, enfermeiro, parteira, amigos, família ou outra pessoa.*
Q_144	*Você recebeu ajuda de outra pessoa para interromper esta gravidez?*
Q_145	*Você recebeu ajuda de um* ***médico*** *para interromper esta gravidez?*

Q_146	*Você recebeu ajuda de um* enfermeiro, enfermeira, assistente de enfermagem ou uma parteira *para interromper esta gravidez?*
Q_147	*Você recebeu ajuda de um* ***farmacêutico ou outra pessoa na farmácia*** *para interromper esta gravidez?*
Q_148	*Você recebeu ajuda* ***de alguém de outro tipo de estabelecimento comercial ou no mercado*** *para interromper esta gravidez?*
Q_149	*Você recebeu ajuda de um* ***trabalhador comunitário de saúde*** *para interromper esta gravidez?*
Q_150	*Você recebeu ajuda* ***de amigos ou amigas*** *para interromper esta gravidez?*
Q_151	*Você recebeu ajuda de* ***uma pessoa da família*** *para interromper esta gravidez?*
Q_152	*Você recebeu ajuda de* ***um médico tradicional ou curandeiro*** *para interromper esta gravidez?*
Q_153	*Você recebeu ajuda de* ***uma parteira tradicional*** *para interromper esta gravidez?*
Q_154	*Você recebeu ajuda de* ***um professor*** *para interromper esta gravidez?*
Q_155	*Você recebeu ajuda de* ***uma outra pessoa*** *para interromper esta gravidez?*
INTRO_156	*Existem muitos locais onde alguém pode tentar interromper uma gravidez, por exemplo em casa ou num outro lugar como farmácia, unidade de saúde ou consultório médico.*
Q_156	*Você tentou interromper esta gravidez* ***em sua casa****?*
Q_157	*Você tentou interromper esta gravidez* ***numa farmácia****?*
Q_158	*Você tentou interromper esta gravidez* em um outro estabelecimento comercial?
Q_159	*Você tentou interromper esta gravidez* ***numa clínica privada****?*
Q_160	
Q_161	*Você tentou interromper esta gravidez* ***numa unidade de saúde pública****?*
Q_162	*Você tentou interromper esta gravidez* ***num consultório médico?***
Q_163	*Você tentou interromper esta gravidez na* ***casa de uma outra pessoa?***
Q_164	*Você tentou interromper esta gravidez* ***em outro lugar?***
INTRO_165	*As próximas perguntas são sobre o atendimento durante a sua permanência neste hospital.*
Q_165	Durante a sua internação aqui, *você recebeu explicações sobre os cuidados e o tratamento que ia receber?*
Q_166	*Você podia fazer perguntas durante o exame e o tratamento?*
Q_167	Você acha que foi informada adequadamente pela pessoa que lhe atendeu sobre as decisões tomadas durante o seu tratamento?
Q_168	*Você sentiu alguma ansiedade ou* stress *durante a sua permanência no hospital?*
Q_169	Você poderia falar para o médico ou enfermeiro que te atendeu que estava se sentindo ansiosa ou estressada?
Q_170	Quando você falou para o médico ou para o enfermeiro que estava se sentindo ansiosa ou estressada, ele ou ela te ofereceu ajuda adicional para melhorar a ansiedade ou o *stress*?
Q_171	*Você acha que as suas escolhas e preferências foram respeitadas durante a internação no hospital?*
Q_172	Você foi tratada com gentileza?
Q_173	Você recebeu remédios para dor durante a sua internação?
Q_174	Os remédios ajudaram a melhorar a dor?
INTRO_175	As próximas perguntas serão sobre o quão satisfeita você ficou com o seu tratamento durante a internação neste hospital. *Para essas perguntas, vamos usar* essas 5 caras *para você escolher uma se você ficou "Muito Satisfeita", "Satisfeita", "Nem Satisfeita, Nem Insatisfeita", "Insatisfeita" ou "Muito Insatisfeita".*
Q_175	*Qual é o seu nível de satisfação com os serviços que recebeu no hospital?*
Q_176	*Qual é o seu nível de satisfação com a privacidade durante o exame e tratamento?*
Q_177	*Qual é o grau de satisfação com relação ao tempo que você teve que esperar para ser atendida por um profissional de saúde nesta instituição?*
Q_178	*Qual é o grau de satisfação com o valor que pagou do seu bolso para os serviços que recebeu?*
Q_179	*Qual é o grau de satisfação com as informações sobre saúde que recebeu dos profissionais de saúde?*
INTRO_180	*Obrigada por ter respondido as perguntas sobre satisfação. As perguntas restantes têm respostas SIM ou NÃO.*
Q_180	*Você diria a uma outra mulher que estivesse nas mesmas condições que você, que viesse a este hospital para ser atendida?*
INTRO_181	*Existem muitos métodos diferentes* para prevenir a gravidez. Alguns desses métodos incluem: esterilização como a laqueadura, DIU, implante, injeção, pílulas, adesivos, anel vaginal ou métodos de barreira como a camisinha.
Q_181	*Você recebeu informação sobre como prevenir uma gravidez durante a sua permanência no hospital?*
Q_182	Caso você não tenha recebido informação sobre maneiras de prevenir a gravidez, você gostaria de receber informações sobre os diferentes métodos disponíveis?
INTRO_183	Existem muitos métodos diferentes para prevenir a gravidez. *Um deles é a esterilização que é permanente. Um exemplo da esterilização é* ligar as tubas da mulher, também chamado de laqueadura. *Outro exemplo é* cortar ou amarrar os tubos que carregam os espermatozoides do parceiro, também chamado de vasectomia.
Q_183	Você foi submetida a um procedimento de esterilização como a ligadura das tubas ou laqueadura?
Q_184	Você foi submetida a esta ligadura tubária ou laqueadura durante esta internação no hospital?
Q_185	*A esterilização foi a sua opção preferida de método contraceptivo?*
INTRO_186	Como mencionamos anteriormente, existem diferentes métodos para prevenir a gravidez. Alguns desses métodos incluem: DIU, implante, injeção, pílulas, adesivos, anel vaginal ou métodos de barreira como a camisinha. À*s vezes as mulheres pedem algum método contraceptivo para evitar a gravidez no futuro.*

	Gostaríamos de saber qual teria sido o seu método preferido, mesmo que você não tenha recebido este método.
Q_186	Você solicitou um DIU nesta internação no hospital?
Q_187	Você solicitou um implante nesta internação no hospital?
Q_188	Você solicitou uma injeção nesta internação no hospital?
Q_189	Você solicitou pílula anticoncepcional nesta internação no hospital?
Q_190	Você solicitou um adesivo nesta internação no hospital?
Q_191	Você solicitou um anel vaginal nesta internação no hospital?
Q_192	Você solicitou um método de barreira como camisinha nesta internação no hospital?
INTRO_193	Às vezes as mulheres recebem um método anticoncepcional ou uma prescrição de anticoncepcional para levar para casa após a internação para evitar futuras gravidezes.
Q_193	*Você vai para a casa com algum método contraceptivo ou com uma receita médica de algum método?*
Q_194	Isto é porque você já não está mais tendo um relacionamento?
Q_195	Isto é porque você tem receio dos efeitos colaterais?
Q_196	*Isto é porque quer engravidar de novo?*
Q_197	*Isto é porque o seu parceiro não concorda?*
Q_198	*Você já começou a usar este método contraceptivo?*
Q_199	*Você vai para a casa com:* ***um DIU?***
Q_200	*Você vai para a casa com:* ***implante?***
Q_201	*Você vai para a casa com:* ***uma injeção?***
Q_202	*Você vai para a casa com:* pílulas anticoncepcionais?
Q_203	*Você vai para a casa com:* ***adesivo?***
Q_204	*Você vai para a casa com:* ***anel vaginal?***
Q_205	*Você vai para a casa com:* ***camisinha?***
THANKS	*Muito obrigada pela sua participação!*<br>*Estamos muito gratos por seu apoio neste estudo importante.*<br>*Por favor, informe o assistente de pesquisa que você já terminou.*
G_01	*Você disse que não quer responder esta pergunta.*<br>*Lembre-se que todas as suas respostas são confidenciais, não serão* compartilhadas ou mostradas para ninguém, *e são muito importantes para nós.*<br>Você pode sempre escolher uma outra resposta. *Ou pode passar para a próxima pergunta.*
G_02	*Você disse que quer parar com a entrevista agora.*<br>*Lembre-se que todas as suas respostas são confidenciais, não serão compartilhadas com ninguém, e são muito importantes para nós.*<br>*Se você decidir terminar a entrevista agora, não poderá responder mais nenhuma pergunta.*<br>*Tem certeza que quer terminar a entrevista agora? Para SIM, PRESSIONE 1. Para NÃO, PRESSIONE 2.*
R1-YES	*Sim*
R2-NO	*Não*
R3-VS	*Muito satisfeita*
R4-S	*Satisfeita*
R5-N	*Nem satisfeita nem insatisfeita*
R6-D	*Insatisfeita*
R7-VD	*Muito insatisfeita*
RG-01	*Para SIM, PRESSIONE 1.*<br>*Para NÃO, PRESSIONE 2.*
RG-02	*Para Muito satisfeita, PRESSIONE 1.*<br>*Para Satisfeita, PRESSIONE 2.*<br>*Para Nem Satisfeita, Nem Insatisfeita, PRESSIONE 3.*<br>*Para Insatisfeita, PRESSIONE 4.*<br>*Para Muito Insatisfeita, PRESSIONE 5.*

Note: adapted from Barbosa Filho et al (2016).

Box S2 World Health Organization *Multi-Country Survey on Abortion* (MCS-A): participating institutions.

FEDERATIVE UNIT	INSTITUTIONS
Rondônia	Hospital Municipal de Ji-Paraná<br>Hospital Regional Adamastor Teixeira de Oliveira<br>Maternidade Mãe Esperança
Maranhão	Hospital Universitário da UFMA<br>Hospital Regional Materno Infantil de Imperatriz<br>Complexo Hospitalar Materno Infantil Do Maranhão<br>Maternidade de Alta Complexidade do Maranhão<br>Maternidade Carmosina Coutinho - Caxias<br>Hospital Materno Infantil - Barra Do Corda<br>Santa Casa de São Luís
Federal District	Hospital Regional de Ceilândia<br>Hospital Regional de Samambaia<br>Hospital Regional de Gama (HRG)<br>Hospital Materno Infantil de Brasília<br>Hospital Regional do Paranoá (HRPA)<br>Hospital Regional de Santa Maria (HRSM)<br>Hospital Regional de Planaltina (HRPL)<br>Hospital Regional Asa Norte (HRAN)<br>Hospital Regional de Taguatinga (HRT)<br>Hospital Regional de Sobradinho (HRS)
